# Supplementary material for: Polly Wants a Genome: The Lack of Genetic Testing for Pet Parrot Species
Source: Genes (Basel). 2021 Jul 20;12(7):1097. doi: 10.3390/genes12071097 (PMC8307168; doi:10.3390/genes12071097)
Supplement: Supplementary file 1 [file genes-12-01097-s001.zip › genes-1252234-supplementary.pdf]

Supplementary file S1

List of parrot species with regularly occurring plumage colour variations.

European Association of Zoos and Aquaria - Parrot Taxon Advisory Group: Simon Bruslund, Marlow Birdpark, chair

Common name	Scientific name
Cockatiel	*Nymphicus hollandicus*
Red-tailed Black-cockatoo	*Calyptorhynchus banksii*
Galah	*Eolophus roseicapilla*
Grey Parrot	*Psittacus erithacus*
Brown Parrot	*Poicephalus meyeri*
Senegal Parrot	*Poicephalus senegalus*
Grey-hooded Parakeet	*Psilopsiagon aymara*
Mountain Parakeet	*Psilopsiagon aurifrons*
Barred Parakeet	*Bolborhynchus lineola*
Monk Parakeet	*Myiopsitta monachus*
Dusky Parrot	*Pionus fuscus*
Blue-headed Parrot	*Pionus menstruus*
White-crowned Parrot	*Pionus senilis*
Southern Festive Amazon	*Amazona festiva*
Cuban Amazon	*Amazona leucocephala*
Red-lored Amazon	*Amazona autumnalis*
Yellow-headed Amazon	*Amazona oratrix*
Yellow-naped Amazon	*Amazona auropalliata*
Yellow-crowned Amazon	*Amazona ochrocephala*
Turquoise-fronted Amazon	*Amazona aestiva*
Orange-winged Amazon	*Amazona amazonica*
Mexican Parrotlet	*Forpus cyanopygius*
Green-rumped Parrotlet	*Forpus passerinus*
Turquoise-winged Parrotlet	*Forpus spengeli*
Blue-winged Parrotlet	*Forpus xanthopterygius*
Spectacled Parrotlet	*Forpus conspicillatus*
Pacific Parrotlet	*Forpus coelestis*
Black-headed Parrot	*Pionites melanocephalus*
Green-thighed Parrot	*Pionites leucogaster*
Maroon-bellied Parakeet	*Pyrrhura frontalis*
Pearly Parakeet	*Pyrrhura lepida*
Green-cheeked Parakeet	*Pyrrhura molinae*
Maroon-tailed Parakeet	*Pyrrhura melanura*
Burrowing Parrot	*Cyanoliseus patagonus*
Hyacinth Macaw	*Anodorhynchus hyacinthinus*
Orange-fronted Parakeet	*Eupsittula canicularis*

Dusky-headed Parakeet	*Aratinga weddellii*
Nanday Parakeet	*Aratinga nenday*
Sun Parakeet	*Aratinga solstitialis*
Jandaya Parakeet	*Aratinga jandaya*
Blue-winged Macaw	*Primolius maracana*
Blue-and-yellow Macaw	*Ara ararauna*
Military Macaw	*Ara militaris*
Great Green Macaw	*Ara ambiguus*
Scarlet Macaw	*Ara macao*
Red-and-green Macaw	*Ara chloropterus*
Northern Red-shouldered Macaw	*Diopsittaca nobilis*
Blue-crowned Parakeet	*Psittacara acuticaudatus*
Mitred Parakeet	*Psittacara mitratus*
Red-rumped Parrot	*Psephotus haematonotus*
Bluebonnet	*Northiella haematogaster*
Mulga Parrot	*Psephotellus varius*
Crimson Rosella	*Platycercus elegans*
Pale-headed Rosella	*Platycercus adscitus*
Eastern Rosella	*Platycercus eximius*
Swift Parrot	*Lathamus discolor*
Ouvea Parakeet	*Eunymphicus uvaeensis*
Yellow-crowned Parakeet	*Cyanoramphus auriceps*
Red-crowned Parakeet	*Cyanoramphus novaezelandiae*
Bourke's Parrot	*Neopsephotus bourkii*
Blue-winged Parrot	*Neophema chrysostoma*
Elegant Parrot	*Neophema elegans*
Turquoise Parrot	*Neophema pulchella*
Scarlet-chested Parrot	*Neophema splendida*
Papuan Lorikeet	*Charmosyna papou*
Stella's Lorikeet	*Charmosyna stellae*
Musk Lorikeet	*Glossopsitta concinna*
Chattering Lory	*Lorius garrulus*
Black-capped Lory	*Lorius lory*
Violet-necked Lory	*Eos squamata*
Coconut Lorikeet	*Trichoglossus haematodus*
Rainbow Lorikeet	*Trichoglossus moluccanus*
Red-collared Lorikeet	*Trichoglossus rubritorquis*
Scaly-breasted Lorikeet	*Trichoglossus chlorolepidotus*
Budgerigar	*Melopsittacus undulatus*
Double-eyed Fig-parrot	*Cyclopsitta diophthalma*
Vernal Hanging-parrot	*Loriculus vernalis*
Blue-crowned Hanging-parrot	*Loriculus galgulus*

Rosy-faced Lovebird	*Agapornis roseicollis*
Fischer's Lovebird	*Agapornis fischeri*
Yellow-collared Lovebird	*Agapornis personatus*
Nyasa Lovebird	*Agapornis lilianae*
Black-cheeked Lovebird	*Agapornis nigrigenis*
Superb Parrot	*Polytelis swainsonii*
Regent Parrot	*Polytelis anthopeplus*
Princess Parrot	*Polytelis alexandrae*
Australian King-parrot	*Alisterus scapularis*
Red-winged Parrot	*Aprosmictus erythropterus*
Eclectus Parrot	*Eclectus roratus*
Grey-headed Parakeet	*Psittacula finschii*
Slaty-headed Parakeet	*Psittacula himalayana*
Blossom-headed Parakeet	*Psittacula roseata*
Plum-headed Parakeet	*Psittacula cyanocephala*
Red-breasted Parakeet	*Psittacula alexandri*
Alexandrine Parakeet	*Psittacula eupatria*
Rose-ringed Parakeet	*Psittacula krameri*
